# Middle Ear Schwannoma

**DOI:** 10.1590/S1808-86942010000500025

**Published:** 2015-10-22

**Authors:** Jorge Luis Roig O. R., Jose Luis Roig-Ocampos F., Daniel Poletti Serafini, Otacilio Lopez Filho

**Affiliations:** 1 Assistant Professor of ENT - Medical Sciences School - Universidade Nacional de Assunção, ENT assistant physician; 2 Full Professor of ENT - Medical Sciences School - Universidade Nacional de Assunção, Chefe do Serviço; 3 Resident Physician - Hospital General Universitário Gregorio Marañon Madrid; 4 Full Professor of Otorhinolaryngology - Medical Sciences School Santa Casa de São Paulo

**Keywords:** ear neoplasms, facial nerve, neurilemmoma

## INTRODUCTION

Of the benign tumors of the middle ear, the paraganglioma is the most common, and the facial nerve schwannoma comes in second place^1^.

Potentially, middle ear schwannomas may stem not only from the facial nerve, but also from its side branches: the chorda tympani; the stapedial nerve; the tympanic branch of the glossopharyngeal nerve and the auricular branch of the vagus nerve^2^. We present here the case of a patient with a middle ear schwannoma of possible chorda tympani origin.

## CASE REPORT

Male, 55 year-old patient complaining of left ear tumor with six months of evolution which in the last 15 days started to hurt. He also reported hypoacusis on the same side for 7 years. Upon physical exam we found a polypoid tumor on the left ear which came out through the external ear canal, of soft consistency and painless (photo 1). There was no facial nerve involvement. Audiological tests showed moderate mixed hypoacusis with a good cochlear reserve in the middle and lower frequencies. A contrasted CT scan showed a radiopaque image which occupied the entire external ear canal (EEC), the tympanic and mastoid cavities. The EEC was enlarged with irregular bone outlining (Photos II and III). We biopsied the lesion and the pathology exam reported Schwannoma (Photo IV).

**Figure:**
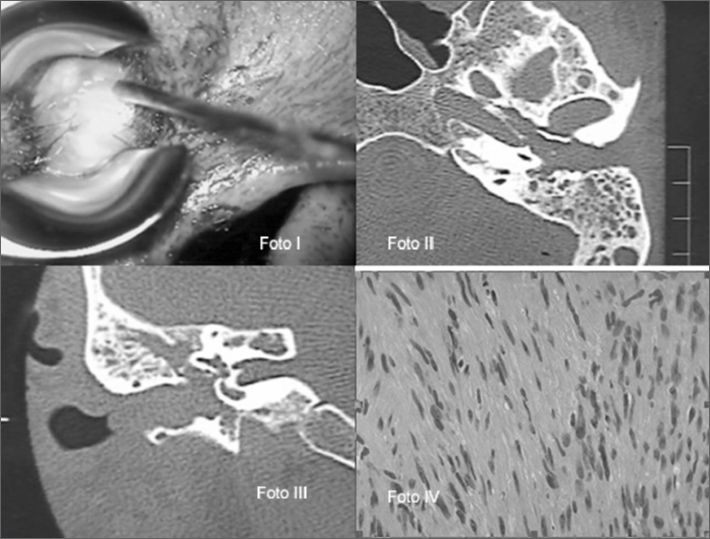
**Photo.** I: Polypoid tumor of the left ear coming out of the EEC. Photos II and III: Contrasted ear CT scan, axial and coronal views, showing lesion extension. Photo IV: Pathology study of the patient with middle ear schwannoma. Proliferation of spindle-shaped cells without atypia, suggestive of schwannoma.

During surgery we noticed a sessile tumor occupying the entire lumen of the EEC. The lesion seemed to have originated in the middle ear, shifting the EEC skin on the postero-superior region. During tumor shifting, we noticed a purulent oozing coming from the middle ear. We then performed a mastoidectomy, aticotomy and we brought the canal wall down as much as possible and, later on, after we had apparently removed the entire tumor, we did a type III tympanoplasty. The tumor involved the hypotympanum, the mesotympanum, aticoantral and peritubal regions. Among other findings, we found an erosion of the long arm of the incus, the stapes superstructure was preserved and we did not notice tumor adhering to the promontory, and the tympanic portion of the facial canal was intact. The patient evolved favorably with functional integrity of the facial nerve with preserved audiometric thresholds. He is currently in the fourth year of follow up, with a favorable outcome.

## DISCUSSION

We suppose the schwannoma came from the chorda tympani because of its anatomical location, facial nerve integrity on the tympanic-mastoid portion and the lack of adherence to the promontory.

Since 1966 until today, there are five reports in the world literature of cases of schwannoma associated with the chorda tympani nerve. The main complaint seen in the papers studies^3^, ^4^, ^5^ was hearing loss; there were also ear fullness, otorrhea and otalgia; we stress the absence of paralysis or facial nerve paresis in all the cases. Our patient also came to us because of hearing loss and otalgia.

In most of the cases, upon otoscopy, the lesion aspect corresponded to a bulging in the postero-superior region of the tympanic membrane or the EEC posterior wall, differently from our patient, whom had a polypoid tumor covered by skin and filling up the entire EEC, bulging out through the canal. There is a report of a cystic tumor which totally occluded the canal and which corresponded to an EEC schwannoma^6^.

CT scan and MRI are tests which enable to define tumor extension and the very nature of the lesion. It would be ideal to count on both studies and, depending on the clinical case, even refrain from biopsing.

In most of the cases published, the surgery was conservative because of the small size of the tumor. In our case, thanks to the large tumor extension, we did a canal wall down mastoidectomy and type III tympanoplasty.

## FINAL REMARKS

The middle ear schwannoma, stemming from the collateral branches of the facial nerve is uncommon, but should be considered in the differential diagnosis of middle ear tumors. Biopsy is a risky procedure, but it was the main diagnostic method in most of the cases published. CT scan and MRI are useful to assess tumor extension and recognize the nature of the lesion. Ultimate treatment is surgical removal, as conservative as possible.
